# Commensal Bacteria Modulate Innate Immune Responses of Vaginal Epithelial Cell Multilayer Cultures

**DOI:** 10.1371/journal.pone.0032728

**Published:** 2012-03-07

**Authors:** William A. Rose, Chris L. McGowin, Rae Ann Spagnuolo, Tonyia D. Eaves-Pyles, Vsevolod L. Popov, Richard B. Pyles

**Affiliations:** 1 Department of Microbiology and Immunology, University of Texas Medical Branch, Glaveston, Texas, United States of America; 2 Department of Pathology, University of Texas Medical Branch, Glaveston, Texas, United States of America; 3 Department of Pediatrics, University of Texas Medical Branch, Galveston, Texas, United States of America; Charité-University Medicine Berlin, Germany

## Abstract

The human vaginal microbiome plays a critical but poorly defined role in reproductive health. Vaginal microbiome alterations are associated with increased susceptibility to sexually-transmitted infections (STI) possibly due to related changes in innate defense responses from epithelial cells. Study of the impact of commensal bacteria on the vaginal mucosal surface has been hindered by current vaginal epithelial cell (VEC) culture systems that lack an appropriate interface between the apical surface of stratified squamous epithelium and the air-filled vaginal lumen. Therefore we developed a reproducible multilayer VEC culture system with an apical (luminal) air-interface that supported colonization with selected commensal bacteria. Multilayer VEC developed tight-junctions and other hallmarks of the vaginal mucosa including predictable proinflammatory cytokine secretion following TLR stimulation. Colonization of multilayers by common vaginal commensals including *Lactobacillus crispatus*, *L. jensenii*, and *L. rhamnosus* led to intimate associations with the VEC exclusively on the apical surface. Vaginal commensals did not trigger cytokine secretion but *Staphylococcus epidermidis*, a skin commensal, was inflammatory. Lactobacilli reduced cytokine secretion in an isolate-specific fashion following TLR stimulation. This tempering of inflammation offers a potential explanation for increased susceptibility to STI in the absence of common commensals and has implications for testing of potential STI preventatives.

## Introduction

The human vaginal mucosa is composed of a non-keratinized stratified squamous epithelium that forms a natural barrier to pathogens [1], [2]. This specialized epithelium has distinctive architectural features including basal cells serving as progenitors for the apical layers that are often anucleate, elongated and frequently are sloughed into the vaginal cavity [3], [4]. The intermediate cell layers contain substantial amounts of glycogen and mucins that localize to vacuoles also present in basal cells [2], [4], [5]. Also, tight junction complexes are a hallmark of the vaginal mucosa [6]. Under specific conditions, the permeability of these complexes is altered to permit passage of innate immune effector molecules secreted by the vaginal epithelial cells (VEC).

The commensal microflora have been increasingly recognized as an important component of the vaginal mucosal defense against STI [7]. Several different species of commensal bacteria including species from the genera Lactobacillus, Corynebacterium, Prevotella and Peptostreptococcus have been identified commonly in vaginal specimens [7], [8], [9], [10], [11]. These commensal organisms are intimately associated with VEC and play crucial but poorly defined roles in vaginal health [12]. In healthy women, the most prevalent commensal Lactobacilli are *L. crispatus*, *L. iners*, *L. jensenii and L. rhamnosus* [7], [11]. Interestingly, *L. iners* also was associated with recovery states following cessation of antimicrobial therapies [10], [13]. Lactobacilli have several important roles for maintaining a healthy vaginal environment including the constitutive production of antimicrobial agents and reducing the extracellular pH of the vaginal mucosa through lactic acid production [7]. It is believed that commensal organism metabolism of the abundant glycogen stores present in VEC in vivo facilitates the symbiotic relationship helping to provide a uniform physical barrier against pathogen adhesion and establishment of infection [2], [4], [7], [14], [15]. Clinically, the loss of vaginal Lactobacilli due to antibiotic therapy, douching, sexual activity, pathologies or other factors is associated with increased susceptibility to subsequent infection [7], [12], [16], [17]. It is of substantial importance then that a relevant and effective model system be developed to allow study of the molecular interactions between human VEC and a microbiome.

Several promising topical agents designed to reduce or prevent experimental STI transmission have proven successful in clinical trials [18], [19], [20]. The previous clinical failures and inadequate predictions of a topical compound's potential may have been precipitated by unintended alterations of the vaginal bacterial ecology that abrogate the protective effect of commensal bacteria. We posit that such alterations also may impact any commensal-associated modulation of innate immune responses elicited by VEC following vaginal application of topical therapies. A human culture system that addresses the impact of common vaginal commensals should help to define the role of these bacteria in vaginal defense and allow for better predictions of clinical outcomes and priorities for clinical testing of promising topical preventative compounds. To this end, we have generated a highly reproducible system, utilizing immortalized cells from 3 donors [21], that form multilayer VEC cultures with an apical air interface modeling the surface of the vaginal mucosa. These multilayer VEC were architecturally similar to the human vaginal mucosa and supported sustained apical colonization by selected common commensal bacteria. Using these cultures we have shown that lab-adapted and clinical isolates of *L. crispatus*, *L. jensenii* and *L. rhamnosus* play an active role in the modulation of the magnitude of cytokine responses in patterns that are distinct from other *Lactobacillus spp* or other less common vaginal bacteria species. This is the first direct evidence for a molecular contribution of Lactobacilli to vaginal health and suggests a much broader role for these bacteria in the dynamic functions of this mucosa. Further study of this model system will allow the identification of the key molecular interactions between the commensal bacteria and the human VEC that lead to enhanced vaginal health and increased resistance to STI. Such studies should reveal novel probiotic approaches to limit infections by human pathogens including HIV.

## Results

### Immortalized human vaginal epithelial cells (VEC) formed multilayer cultures consistent with stratified squamous epithelia

**Figure 1:**
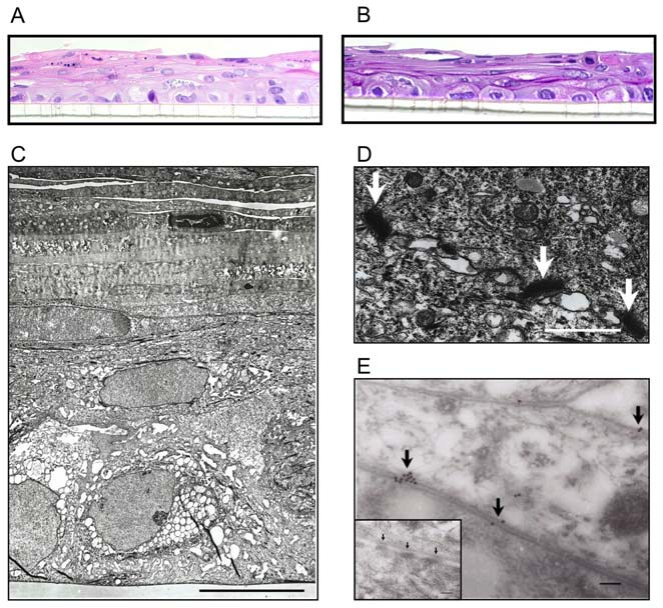
Human immortalized VEC differentiated into multilayers with air-interfaces morphologically similar to vaginal stratified squamous epithelium. **A**. A representative H&E (400×) staining of a d 9 V19I VEC multilayer 5 um section that illustrates the formation of multiple cell layers. The transwell porous membrane can be seen at the bottom of the micrograph. **B**. PAS staining of a 5 uM section through a VEC multilayer illustrated the substantial amounts of glycogen produced. VEC grown in standard format did not stain by PAS (data not shown). **C**. A representative transmission EM image of a d 9 V19I VEC multilayer culture (>7 layers) including the transwell porous support (bottom) and the apical anucleate layers sloughing from the top surface (bar = 10 uM). The micrograph illustrates the intracellular structures and the formation of tight junctions indicating full polarization and differentiation. **D**. Tight junction complexes (white arrows) were observed between the plasma membranes of VEC within 7–9 d of plating (bar = 100 nm) and were confirmed, **E**., by immunogold labeling for occludin (black arrows show gold particle deposition; bar = 100 nM). Inset panel: A secondary antibody control transmission EM showed no labeling at the tight junction complexes (black arrows, bar = 100 nM).

To enhance the quality of human vaginal culture models, immortalized VEC from one of three donors were seeded into transwell culture inserts. After 48 h, the growth medium was removed from the apical chamber forming an air-interface. Trans-epithelial electrical resistance (TEER) monitoring of VEC multilayers, temporarily covered in PBS, significantly (p<0.001) increased by day 7–9 (avg. = 228.9±6.4 Ωcm^2^) relative to TEER values at seeding (day 0 avg. = 41.4±0.3 Ωcm^2^) and were maintained through d14. To address histological characteristics, established multilayers (day 9) on the filter support were fixed and then carefully excised from the inserts for paraffin embedding. Hematoxylin and eosin (H&E) stained cross sections showed that VEC from each of the 3 donors formed multiple cell layers with nucleated cells at the basolateral surface and elongated, often anucleate cells at the apical surface (Figure 1A). These observations were consistent with histological sections obtained from human vaginal mucosal biopsies [3], [5]. Periodic acid Schiff (PAS) staining of sequential multilayer culture sections showed substantial glycogen stores were present at intracellular sites (Figure 1B) similar to those observed in normal vaginal tissue [2], [4], [6]. In conjunction with confocal and transmission electron microscopy (EM) the H&E staining images reproducibly indicated that the VEC multilayers were composed of 7–12 cell layers (avg. thickness of 48±9 um) 7–9 d after seeding.

Transmission EM evaluations of day 9 cultures illustrated that VEC contained large numbers of vacuoles (Figure 1C). Basal and intermediate cells contained large vacuoles while smaller vacuoles were observed in the elongated, often anucleate apical cells (Figure 1C) that labeled positively for glycogen (Figure 1B) or mucins (data not shown). TEM also indicated the presence of tight junction complexes between the plasma membranes of cells (Figure 1D). Immunogold-labeling for the tight junction protein occluding [22], confirmed the identity of these structures (Figure 1E). Secondary antibody alone did not label the structures in subsequent sections (Figure 1E, insert). With regard to reproducibility, VEC multilayers established with cells from any of the three donors produced indistinguishable micrographs (data not shown) indicating that immortalized VEC under our culture conditions reproducibly formed multilayer cultures with apical air-interfaces that architecturally modeled the human vaginal mucosa.

### VEC multilayer cultures responded to selected TLR agonists

To evaluate innate responses of VEC multilayers, cultures were treated apically with a panel of TLR agonists previously tested in standard culture format [21]. Specifically, PIC (TLR3), FSL-1 (TLR2/6), FLAG (TLR5) and PGN (TLR2) were tested because they are recognized by the most abundantly expressed TLRs in human VEC [21]. VEC multilayers established with cells from each of three donors produced similar TLR agonist-induced cytokine profiles consistent with our previous findings in standard culture formats [21]. Application of PIC, a synthetic double-stranded RNA that is recognized by TLR3, resulted in significant (p<0.05) secretion of IL-1ra, IL-6, IL-8, GM-CSF, MIP-1b, RANTES and TNFa at 6 h post application compared to parallel cultures treated with vehicle only (Table 1). FSL-1, a bacterial-derived TLR2/6 agonist, elicited a distinct cytokine profile with significant (p<0.05) increases in IL-6, IL-8, IL-12(p70), G-CSF, GM-CSF, MIP-1b, RANTES and TNFa relative to vehicle alone (Table 1). The addition of other bacterial-derived agonists, recognized through different TLR, led to significant (p<0.05) elaboration of IL-6, IL-8, G-CSF, MIP-1b and TNFa for FLAG (TLR5) and IL-1b, IL-1ra and TNFa for PGN (TLR2) (Table 1). The observed agonist-specific cytokine profiles were consistent with our previously reported findings from standard culture formats [21] and indicated that multilayer VEC responded to TLR agonists. Of the 4 tested agonists, PIC and FSL-1 produced the most robust cytokine induction with common significant increases in IL-6, IL-8 and TNFa. For the remaining studies, results for cytokines induced by PIC or FSL-1 will be presented.

**Table 1:** TLR agonists induced specific cytokine profiles in VEC multilayer cultures.

Cytokine^b^	Medium ctrl	TLR Agonists^a^			
		PIC	FSL-1	FLAG	PGN
**IL-1b**	12±3	18±1	11±1	9±2	58±9^e^
**IL-6**	11±1	1057±63^e^	155±17^d^	137±7^e^	9±4
**IL-8**	96±11	24863±8773^c^	24867±8769^c^	19258±2566^d^	647±206
**IL-12(p70)**	40±4	42±3	57±4^c^	42±4	34±5
**TNFa**	18±2	460±3^e^	263±14^e^	434±33^e^	29±2^d^
**G-CSF**	58±16	46±4	524±29^e^	1002±88^e^	13±5
**GM-CSF**	9±1	25±2^e^	23±4^d^	39±7^d^	5±1
**MIP-1b**	5±2	41±1^e^	138±3^e^	28±7^d^	3±1
**RANTES**	55±21	313±11^e^	302±2^e^	78±10	62±15
**IL-1ra**	9844±1943	42757±3225^e^	13060±2479	8198±1313	86236±5018^e^

a PIC (0.1 mg/mL), FSL-1 (0.1 ug/mL), FLAG (5 ug/mL), PGN (0.1 mg/mL) or a medium control (ctrl, 10 uL) was added to the apical surface of V11I d 10 multilayer cultures. Apical sampling (100 uL) was completed 6 h later for cytokine analyses.

b Selected pro-inflammatory cytokines (top), chemokines (middle) and anti-inflammatory (bottom) cytokines are presented out of the 27 tested using the multiplex array system. Data are the mean ± SEM (pg/mL) of 3 replicates from a representative experiment of 3 independent experiments.

c p<0.05 compared to Medium ctrl (Student's t-test).

d p<0.01 compared to Medium ctrl (Student's t-test).

e p<0.001 compared to Medium ctrl (Student's t-test).

### Commensal bacteria colonized the apical surface of human VEC multilayer cultures

**Figure 2:**
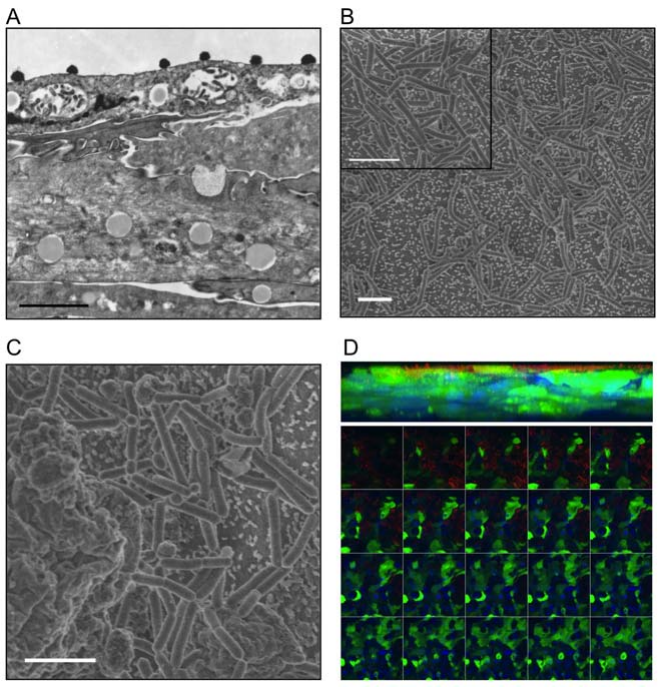
Commensal bacteria colonized the apical surface of air-interface VEC multilayer cultures. **A**. A representative transmission EM micrograph showing the top half of a VEC multilayer culture 24 h post colonization with *L. jensenii* on the apical surface illustrating the glycogen vesicles contained in the VEC that increased in size and density in the apical most layers of the cultures (bar = 2 um; 14,100×). **B**. Scanning EM micrographs illustrated the apical colonization of *L. jensenii* (24 h) and the intimate associations between bacteria and VEC microvilli present on the surface (bar = 2 um; 3000×). Apparent daughter bacterial cells with shorter length as well as areas of higher bacterial density were observed (inset panel). **C**. Commensal bacteria did not associate with the surfaces of cells that were condensed and apparently sloughing as indicated by their position above the average plane of the apical surface (e.g. cell left side of image; bar = 2 um; 10,000×). **D**. To confirm the location of the Lactobacilli colonizing the VEC multilayers, confocal Z-axis projection (63×; top panel) and optical slices (bottom panels in order) from a GFP-expressing VEC multilayer (green) with DAPI stained nuclei (blue) and antibody labeled *L. jensenii* (red) illustrated exclusive superficial colonization of the apical cell surface. No evidence of bacteria (red labeling) in sections below the apical surface was obtained even after 72 h of colonization.

Having established basic morphological and immunological characteristics of VEC multilayer cultures, the ability of the model to support common vaginal commensal bacteria (*L. crispatus* or *L. jensenii*) colonization was evaluated. As an initial qualitative evaluation of colonization, bacterial adherence as well as potential inappropriate penetration into the VEC multilayers was analyzed via EM and confocal microscopy (Figure 2). Transmission EM analyses confirmed the presence of rod-shaped Lactobacilli (*L. jensenii* shown) tightly associated with VEC exclusively on the apical surface of each multilayer examined (Figure 2A, electron dense rods shown in cross section). These transmission EM micrographs also indicated that apical cells appeared intact and contained multiple glycogen vacuoles, tight junctions and multilayer architecture similar to non-colonized cultures that were seeded at the same time and processed in parallel.

Scanning EM provided additional imagery to illustrate the location and associations of the bacteria with the apical VEC. In most fields, the bacteria were in direct contact with microvilli-like structures on the apical most cells and were distributed relatively evenly across the surface (Figure 2B and C). The average length of *L. jensenii* in contact with VEC, calculated by measuring bacteria from 33 fields, was 1.45 um±0.08 um. Similar sizes were observed for *L. crispatus* that were inoculated onto parallel VEC cultures (data not shown). Interestingly, bacteria were not observed to attach to the VEC that were anucleate with ruffled membranes consistent with a detachment process seen in mucosal sloughing (Figure 2C left side of the micrograph).

Importantly, both transmission and scanning EM showed no penetration of Lactobacilli into deeper layers and no intracellular bacteria were observed in any sections (>50 examined) from colonized multilayers established from all three donors. Apical surface localization was confirmed using GFP-expressing VEC multilayers and confocal microscopy. GFP-VEC were seeded with *L. jensenii*, cultured for 24 or 72 h and then fixed and immunolabeled to localize the bacteria. Optical sectioning through the culture confirmed that the bacteria did not penetrate into the multilayers as evaluated by multiple compiled Z-stacks (Figure 2D top panel). Individual slices showed that bacteria localized to apical surfaces with some pooling in the valleys that formed in each culture (Figure 2D, lower panel).

**Figure 3:**
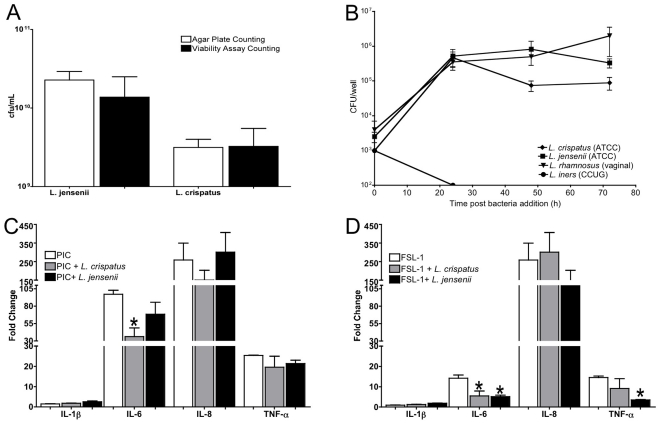
Commensal bacteria colonization kinetics and impact on VEC multilayer cultures. **A.** A higher-throughput broth-based bacterial titration system was validated against standard agar plating and subsequent colony counting. Comparison of the two methods showed no statistical differences in viability quantification (p>0.05, Student's t-test) supporting subsequent use of the broth-based viability assay. Data are the mean ± SEM of duplicate samples from 2 independent experiments. **B.** Replication kinetics and steady state formation of *L. crispatus*, *L. jensenii* or L. *iners* (10^3^ cfu/well) in VEC multilayer cultures are shown over 72 h. *L. iners* viability rapidly decreased and no viable bacteria were observed after 24 h. *L. crispatus* and *L. jensenii* viability significantly (*, p<0.05, Student's t-test) increased after 24 h of culture and was maintained over the study period. Each time point for the selected bacterial species represents the mean ± SEM for triplicate wells of each VEC type (V11I, V12I, V19I) from 2 independent experiments. **C and D**. Colonization of the VEC by *L. crispatus* and *L. jensenii* differently impacted responses to TLR agonists (PIC or FSL-1, respectively) as indicated by the fold change in cytokine levels relative to non-colonized cultures treated in parallel. Data are the mean ± SEM of 3 replicates from a representative experiment out of 3 independent experiments.

The surface area of the multilayer cultures in 96 well format was determined to be 80 mm^2^ representing ∼0.1% of the human vaginal mucosal surface [6]. Based on this calculation, we estimated that an effective model system should be colonized by a bacterial population of ≤10^6^ viable organisms based on reported levels of vaginal *Lactobacilli spp* colonization (10^9^ bacteria) [8]. In preliminary studies, we established that multilayers seeded with 10^3^ viable bacteria created a stable bacterial load of 10^5^ colony-forming units (cfu) within 24 h. To quantify the viable bacterial titers in colonized cultures at later time points, we adapted the standard serial dilution quantification method with agar plates to a higher-throughput, broth-based, 96-well plate format and confirmed its validity by comparison for both *L. jensenii* and *L. crispatus* (ATCC type strains; Figure 3A). Titers were established by observation of bacterial growth in the highest dilution and then calculating the viable bacterial load by multiplying by that dilution factor.

**Figure 4:**
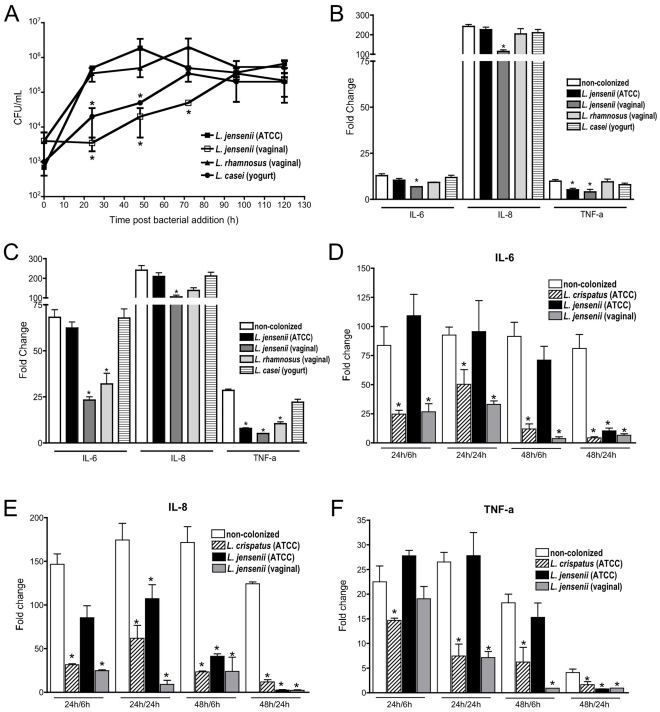
Commensal bacteria colonization from low pass clinical isolates and lab adapted strains showed distinct alterations in TLR agonist-induced cytokine expression. **A**. Colonization kinetics for several vaginal and non-vaginal Lactobacilli are presented over 5 d with statistically delayed growth for several of the isolates relative to the *L. jensenii* ATCC type strain (*p<0.05, ANOVA) providing the opportunity to evaluate the impact of bacterial density on cytokine induction. **B and C.** Data from the standard paradigm indicated that after 24 h of bacterial colonization and 6 h of TLR agonist exposure (FSL-1 or PIC, panels B and C, respectively) showed even reduced titers of the indicated Lactobacilli strains could significantly reduce cytokine induction relative to non-colonized controls treated in parallel (*p<0.05, ANOVA). Colonization with the clinical *L. jensenii* (vaginal) isolate that reached 24 h titers ∼100-fold lower than the lab adapted isolate (ATCC) produced the most robust reduction in cytokine expression with more pronounced effects following PIC exposure (panel C). **D–F.** Given the different kinetics of colonization observed for each isolate we also kinetically tested selected bacteria for impact on PIC cytokine induction following either 24 or 48 h of colonization. TLR agonist exposures also were evaluated at two time points (6 and 24 h) creating four testing conditions both confirming and extending the previous observations. The data are presented as fold change relative to non-colonized cultures treated with PIC in parallel for IL-6 (**D**), IL-8 (**E**) and TNFa (**F**; * p<0.05).

Using this liquid-based quantification system, the viability of *L. crispatus*, *L. jensenii*, *L. rhamnosus* and *L. iners* in air-interface VEC multilayer cultures was evaluated at 24, 48 and 72 h post addition of bacteria. Interestingly, *L. iners* did not viably colonize the multilayers (Figure 3B) indicating an inadequate growth environment. Viable titers of *L. crispatus* and *L. jensenii* (ATCC) and a *L. rhamnosus* clinical isolate increased significantly (p<0.05) over the first 24 h reaching equilibrium for at least an additional 4 d (Figure 3B & 4A). Together the results indicated that VEC multilayer cultures supported long-term colonization by several common vaginal Lactobacilli without observable morphological changes to the VEC multilayer.

The potential impact of commensal colonization on air-interface VEC multilayers was evaluated more specifically by measuring cell health, tight junction integrity and pH changes. *L. crispatus* colonization of d 9 V19I multilayers did not significantly (p>0.05) impact the health of the culture over a 72 h time period (0.2±0.03 optical density by MTT assay) compared to medium only cultures (0.2±0.02). Evaluation of multilayer integrity using TEER measurements showed no significant (p>0.05) difference between *L. crispatus*-colonized cultures (235.3±2.2 Ωcm^2^) and controls at 72 h (235.6±4.7 Ωcm^2^). Similar outcomes were established for cultures colonized with *L. jensenii*. Interestingly, using a pH sensitive indicator material, neither *L. crispatus* nor *L. jensenii* altered the pH of the apical surface of colonized VEC multilayers relative to non-colonized cultures (p>0.05; non-colonized pH 7–7.2; *L. crispatus* colonized pH 6.8–7.0; *L. jensenii* pH 7.0–7.2).

### Colonization with commensal bacteria altered inducible cytokine profiles in human VEC multilayers

The impact of colonization by commensal bacteria on VEC cytokine secretion alone or following recognition of TLR agonists was evaluated. Initial evaluations were performed to identify potential cytokine inductions in response to colonization by *L. crispatus*, *L. jensenii* or, as a control, *Staphylococcus epidermidis*. *S. epidermidis* was selected as a non-vaginal commensal control because it is isolated occasionally from the human vagina [23] but is more commonly a commensal bacterium of the skin. Quantification of secreted cytokines showed that *L. crispatus* or *L. jensenii* colonization of VEC multilayer cultures did not significantly (p>0.05) alter basal levels of cytokine base on comparison to levels detected in non-colonized VEC multilayers (Table 2). Interestingly, colonization of parallel multilayer cultures with *S. epidermidis* resulted in a significant (p<0.05) increase in IL-1b, IL-1ra, IL-8, G-CSF and TNFa (Table 2) relative to non-colonized cultures. These results correlate with clinical observations that *L. crispatus* and *L. jensenii* colonization does not lead to inflammation and in fact may reduce proinflammatory cytokine production [7], [9].

**Table 2:** VEC multilayer cultures selectively responded to non-vaginal commensal bacteria.

Cytokine^b^	Medium ctrl	Commensal Bacteria^a^		
		*L. jensenii*	*L. crispatus*	*S. epidermidis*
**IL-1b**	12±3	7±1	10±1	43±9^c^
**IL-6**	11±1	16±3	15±2	31±13
**IL-8**	96±11	83±6	166±24	1058±107^c^
**IL-12(p70)**	40±4	40±3	34±3	35±5
**TNFa**	22±4	24±3	17±2	131±47^d^
**G-CSF**	58±16	37±7	76±29	131±18^d^
**GM-CSF**	7±1	7±1	8±2	18±4
**MIP-1b**	5±2	6±2	6±2	7±1
**RANTES**	55±21	54±16	51±17	155±73
**IL-1ra**	9844±1943	11453±2083	10343±3372	31653±7806^d^

a *L. jensenii* (1000 cfu), *L. crispatus* (1000 cfu), *S. epidermidis* (1000 cfu) or a medium control (ctrl, 10 uL) was added to the apical surface of V11I d 9 multilayer cultures. Apical sampling (100 uL) was completed 6 h later for cytokine analyses.

b Selected cytokines are presented. Data are the mean ± SEM (pg/mL) of 3 replicates from a representative experiment of 3 independent experiments.

c p<0.001 compared to medium ctrl (Student's t-test).

d p<0.05 compared to medium ctrl (Student's t-test).

To assess the impact of commensal colonization on inflammation, we treated established VEC multilayers with the selected TLR agonists in the context of *L. crispatus* or *L. jensenii* colonization. Specifically, PIC or FSL-1 was added apically to triplicate cultures colonized for 24 h followed by quantification of inflammatory cytokine production. The induction of cytokines was compared to non-colonized VEC multilayer controls processed in parallel. Interestingly, colonization of VEC multilayer cultures with *L. crispatus* was associated with a reduction in selected cytokines induced 24 h after agonist stimulation (Figure 3C and D). PIC induction of IL-6 and IL-8 was reduced significantly (p<0.05) in *L. crispatus* colonized cultures compared to non-colonized cultures (Figure 3C). Surprisingly, *L. jensenii* colonized cultures did not show significant reductions following PIC application although there was an apparent trend. Similarly, the presence of *L. crispatus* or *L. jensenii* on VEC multilayers significantly reduced (p<0.05) IL-6 and TNFa secretion following FSL-1 stimulation. Other reductions in cytokine secretion following PIC or FSL-1 were observed including IL-1ra, IL-12, G-CSF, MIP-1b, and RANTES for *L. crispatus* and to a lesser extent *L. jensenii* (data not shown). Collectively, these results suggested that *L. crispatus* colonization produced a more substantial impact on TLR agonist-induced cytokine elaboration than *L. jensenii*, but both bacteria had capacity to reduce induced inflammatory outcomes.

### A clinical isolate of L. jensenii but not other Lactobacilli spp had pronounced effects on cytokine induction

To address the concern that lab adaptation of the type strain of *L. jensenii* affected its ability to colonize or impact the VEC cultures we isolated several additional clinical *Lactobacilli spp* from vaginal secretions and confirmed their identity and purity through 16 S ribotyping [24]. In addition to a low passage clinical isolate of *L. jensenii*, we also evaluated several *L. rhamnosus* isolates common to the vaginal mucosa. Additionally, we isolated *L. casei* from commercial yogurt to evaluate the behavior of a non-vaginal Lactobacillus species in our VEC culture system.

The clinical isolates of *L. jensenii* and *L. rhamnosus* as well as *L. casei* colonized the VEC multilayers establishing predicted steady state titers by 120 h post inoculation (Figure 4A). Interestingly, the low pass clinical *L. jensenii* isolate colonized cultures more slowly than the *L. jensenii* ATCC type strain with a consistent and significant delay in the establishment of a steady state (figure 4A). This also was the case for *L. casei* but not for the clinical *L. rhamnosus* isolate that exhibited kinetics similar to the lab adapted isolates of *L. crispatus* and *L. jensenii* (Figure 3B and 4A).

The distinct kinetics and potential for altered interactions with the VEC multilayer led us to evaluate the impact of each commensal bacteria on cytokine induction following TLR agonist exposure over time. This approach also addressed the impact of differing bacterial titers on the VEC response to the TLR agonist. For these studies, VEC multilayers were colonized with either the lab adapted *L. jensenii* or the clinical isolates for 24 h prior to addition of FSL-1 (Figure 4B) or PIC (Figure 4C) followed by sampling 6 h later reproducing our previous study design (Figure 3). *L. casei* was included to determine if non-vaginal Lactobacilli had any impact upon inflammation in our culture system. After treatment with either of the TLR agonists, even with 100-fold fewer bacteria, the clinical *L. jensenii* showed significant tempering of cytokine induction relative to the type strain (Figure 4 panels B and C). The *L. rhamnosus* isolate had no appreciable impact on cytokine induction following FSL-1 treatment (Figure 4B) but had significant impact on selected cytokines after PIC exposure (Figure 4C). Finally, the non-vaginal *L. casei* was well tolerated by the VEC but did not impact cytokine responses following treatment with either TLR agonist (Figure 4B and C).

The observation that the clinical *L. jensenii*, even at 100-fold lower titer, significantly tempered the induction of cytokines led us to complete a limited time course evaluation comparing the impact of the type strain and clinical *L. jensenii* isolates on PIC cytokine induction. To confirm and extend the prior results, we also evaluated the impact of the *L. crispatus* type strain. As shown in Figure 4 panels D–F, we colonized VEC for either 24 or 48 h prior to treatment with PIC for 6 or 24 h (24/6, 24/24 or 48/6, 48/24). The results for the *L. jensenii* type strain indicated that despite establishment of steady state titers within 24 h of seeding, the tempering of cytokine induction was more pronounced over time. This suggested that the interaction between bacterial cells and the apical VEC surface elicited molecular influences in the bacteria that, in turn, tempered the cytokine response. Transfer of bacteria from these cultures to fresh cultures required similar amounts of time to impact VEC responses (data not shown). In contrast, the *L. crispatus* type strain had a significant impact on cytokine induction that was evident at all evaluated time points confirming the previous results (Figure 3). These results suggested that any lab adaptations in this isolate were not associated with loss of the ability to establish relationships with the VEC. Finally, these data confirmed that the clinical *L. jensenii* produced the most robust temperance of responses to PIC even at the lowest bacterial titers after 24 h of colonization (Figure 4A). The data for all three bacteria indicate that molecular contributions of the particular isolate during the process of colonization of the VEC was more important to the impact on cytokine induction than actual titer of the commensal organism.

## Discussion

Although the clinical importance of the vaginal microbiome has been well established [7], the molecular basis of the contribution of these bacteria has been understudied because of poor animal and in vitro model systems. Many approaches for modeling the human vaginal mucosa have been developed with differing success but collectively the current systems lack the ability to reproducibly recapitulate the complexity of the stratified squamous epithelium. To date none of the culture systems have created a useful apical surface with an air interface where commensal and pathogenic bacteria can properly colonize. We report a refined transwell culture system using immortalized VEC [21] that created polarized and differentiated multilayers with an apical surface environment adequate for colonization by selected common vaginal Lactobacilli. We report different capabilities of both lab adapted and low passage clinical isolates of common vaginal *Lactobacilli spp*. to colonize and impact these cultures; a novel paradigm for classification of these important bacterial strains. *Lactobacilli spp*. colonization was well tolerated by our VEC multilayers but colonization by *S. epidermidis* led to inflammatory cytokine responses indicating the cultures retained the ability to distinguish common commensal species from transient organisms not normally present in the vaginal microbiome. Although our culture system offered substantial improvements, it was unable to support sustained colonization by the common vaginal commensal *L. iners* suggesting additional refinements are still needed to better support this fastidious organism.

The multilayer air-interfaced VEC culture system facilitated development of a stratified, squamous epithelium with relevant characteristics including basal progenitor cells and anucleate cells at the apical surface that appeared to be separating from the deeper, nucleated layers similar to that seen in vivo (Figure 1). The value of such a culture is substantial because of the distinct differences in vaginal tissues in animal models. For example, the commonly used rabbit vaginal irritation model, that is Food and Drug Administration approved for evaluating the safety of vaginally-applied compounds [25], lacks stratified squamous epithelium and in fact is primarily composed of columnar epithelial cells [26]. Non-human primate, sheep and pig models provide an improved representation of the human vaginal mucosa [27], [28], [29], [30], [31], [32] but experiments involving these large animals are expensive and animal availability is limited. Rodent models also have been used commonly to evaluate the activity and safety of vaginally-applied compounds [30], [33], [34], [35].

Unfortunately, the distinct physiological differences in the vaginal environment of each of the animal models including distinct microbiomes limit their utility to address the impact Lactobacilli common to the human vagina [27], [36], [37], [38]. Zarate and colleagues placed the probiotic *L. paracasei* (CRL 1289) in murine vaginal tracts showing that this probiotic transiently colonized the mouse vagina and reduced titers of uropathogenic *S. aureus* [39]. Utilizing more common vaginal commensals, Jerse and her colleagues also evaluated murine vaginal colonization with *L. jensenii* [40] and in a later report *L. crispatus* [41]. Although colonization by either bacterium was transient after a single application, using two inoculations of *L. crispatus* the group was able to establish sustained vaginal colonization [41]. With this success, it is possible that the mouse could be conditioned to provide a supportive environment for human vaginal commensals but additional work is needed to refine the method for colonization with other Lactobacilli.

Human explant models have been developed using tissues obtained during hysterectomies but have shown intra- and inter-subject variability and are limited to available donors and the amounts of tissue collected [42]. The cultures also are sterile and are relatively short-lived with minimal capacity for follow up studies in the same genetic background. While organotypic cultures generated from primary ectocervical/vaginal cells are commonly and now even commercially available, they are expensive, are limited in supply, are associated with substantial biological variation, lack an air interface and do not include commensal bacteria. Recent advances with bioreactors using rotating wall vessel technology [43] have produced solid alternatives to existing culture systems but lack defined basal and apical chambers and importantly do not include the option for an air interface at the mucosal surface.

Utilizing our refined VEC multilayers we first determined that selected bacterial species colonized rather than replicated in an unrestricted fashion in the cultures. Previous experiments with vaginal commensal bacteria only evaluated the impact of dead bacteria [44] or live bacteria within an 8 h time period [14] and did not provide effective modeling of the bacterial VEC interface. In our colonized VEC multilayer cultures, viable bacteria were recoverable up to at least 5 d post addition of bacteria and importantly they established a steady state of colonization similar to levels predicted by bacterial load estimations from clinical specimens [8]. No impact on cell health and tight junction integrity was observed following bacterial colonization indicating a symbiotic relationship is established between the bacteria and the VEC. In support of symbiosis between the VEC and bacteria, glycogen and mucin containing vesicles were plentiful throughout the VEC layers but higher concentrations in the more apical layers of the cultures were visualized where the bacteria localized (Figure 1B). Such vesicles likely contributed to successful Lactobacilli colonization because glycogen is utilized by Lactobacilli for metabolic functions including the production of lactic acid that contributes to the acidic pH of the vaginal surface [7]. In our studies, each of the *L. jensenii* and *L. crispatus* isolates produced lactic acid when grown axenically (MRS broth culture pH was shifted from 6.0 to less than 4.0 after 18 h of culture). Despite this finding, colonization did not lead to detectable VEC surface pH changes even after multiple approaches were attempted. The lack of a pH shift in the culture environment most probably reflects the substantial buffering capacity of the culture medium in the basal chamber. It is notable that the health of the VEC was negatively impacted when the pH of the medium became acidic in the absence of bacteria.

In addition to different species of commensal bacteria, the observed differential rates of colonization by lab adapted and clinical Lactobacilli isolates led us to study the impact of the bacteria on VEC behavior using multilayers created in 96 well formats. Studies of other Lactobacilli in the context of intestinal mucosal colonization have suggested that these bacteria can create anti-inflammatory signals in host cells with which they associate [45], [46]. We tested the hypothesis that appropriate commensal bacteria might alter innate immune responses as indicated by cytokine production following a controlled insult from a TLR agonist as reported previously [21]. Colonization of VEC multilayers with *L. jensenii* and *L. crispatus* isolates differentially tempered pro-inflammatory outcomes. The lab-adapted *L. jensenii* isolate (ATCC 25258) retained the ability to modulate inflammation but this effect required prolonged colonization of the VEC despite the most robust replication of the bacteria after culture inoculation. This trait was not retained after subculture to another VEC multilayer (data not shown) suggesting that transient bacterial transcriptome and/or proteome changes were responsible for impact on VEC. This is an ongoing area of research that will capitalize on the differences observed between the clinical and lab-adapted strains. In contrast, non-vaginal Lactobacilli failed to alter VEC responses to the tested TLR agonists despite effective colonization indicating that the VEC environment did not create the specific molecular relationships for these isolates.

Establishing molecular mechanisms for the commensal microbiome's contribution to vaginal health is crucial to improved topical drug and vaccine development as well as basic understanding of commensal bacterial contributions to tissue development. Chronic elaboration of pro-inflammatory cytokines has been linked with disruption of the integrity of the vaginal mucosa and activation of HIV in infected individuals [47]. The *S. epidermidis* isolate tested in our study induced an inflammatory cytokine response profile that was similar to that observed for women with bacterial vaginosis (BV) [16], [48], [49], [50]. BV is a polymicrobial condition characterized by replacement of Lactobacilli with anaerobic bacteria [51]. This leads to a state of acute or persistent mucosal inflammation and is associated with increase susceptibility to STI [16], [48], [52]. In the VEC multilayer cultures, Lactobacilli colonization significantly reduced IL-6, IL-8 and TNFa secretion after TLR stimulation supporting the hypothesis that, in vivo, the presence of BV bacteria and the absence of commensals may combine to produce elevated inflammation. Additional model refinements will be necessary to address this hypothesis but the current data are consistent with that conclusion.

The role of commensal bacteria in mucosal health is an area of intense research and recent studies have indicated the remarkable impacts of these bacteria on tissue development, immunity, as well as systemic effects on tissues distant to the sites of colonization [53]. Study of the molecular interactions between commensal bacteria and the vaginal mucosa will likely reveal novel avenues for probiotic development as well as improved systems for vaginally applied compounds to enhance resistance to infections. We believe that our culture system offers an important advance for such experiments as evidenced by these first observations that indicate an inflammatory temperance effect is provided by stable colonization with relevant commensal bacteria. This novel in vitro model is widely adaptable for evaluating other important biological questions such as the impact of commensals on STI acquisition prior to or just after antimicrobial treatment. Others have suggested the use of Lactobacilli probiotics in treatments for a variety of vaginal infections including BV [54] and, through genetic modification of probiotic Lactobacilli strains, specific STI including HIV [55]. It is clear that a delicate balance of bacterial species is associated with normal vaginal health [11], [56] but additional investigations are needed to determine the optimal profile of species and ratios for probiotic treatments to succeed. We believe that this VEC culture system is a reliable, reproducible platform to perform such evaluations. Importantly, our data suggest that testing of vaginally applied compounds in the context of commensal bacteria will provide a more informative and predictive measure of success prior to clinical evaluations.

## Methods

### Air-interface Multilayer Culture Model

Human immortalized VEC from 3 donors (V11I, V12I, V19I) were cultured as described previously [21]. The human VEC multilayer cultures were generated by addition of VEC (1×10^5^ cells) in antibiotic-free, keratinocyte serum-free medium (KSFM; Invitrogen, Carlsbad CA) to the top chambers of 96 well insert system in an angled bottom plate (Becton, Dickinson and Company; (BD) Franklin Lakes, NJ) containing 260 uL of KSFM in the bottom chambers. Cultures were incubated for 24–36 h before the medium from the top chamber was removed to form the air-interface. Medium in each bottom chamber was replaced every other day. Trans-epithelial electrical resistance (TEER; World Precision Instruments, Sarasota, FL) values are reported as the mean of 3 culture inserts ± SEM for each time point. Cultures were considered mature based on TEM, SEM and TEER evaluations at day 7–9 after plating. Prior to the start of any experiment, fresh medium was added to the bottom chamber.

### Bacterial Culture, Colonization of Multilayer Cultures and Viability Quantification

Bacteria were subcultured in De Man, Rogosa, Sharpe (MRS) broth (BD) for *Lactobacillus jensenii* (ATCC 25258) and *Lactobacillus crispatus* (ATCC 33820) or Tryptic Soy (TS) broth (BD) for *Staphylococcus epidermidis* (ATCC 35984) then stored at −80°C in bacterial specific medium with 15% glycerol (Fisher, Pittsburgh, PA). Four different clinical isolates of *Lactobacillus iners* (kindly provided by Bernard Moncla) were subcultured on chocolate agar plates (PML, Wilsonville, OR) in anaerobic conditions using Bio-Bags (BD) then stored at −80°C in Litmus Milk (BD).

VEC multilayer cultures (d 9) apical surfaces were inoculated with log phase bacteria prepared fresh before each study. Frozen bacterial aliquots were thawed into 5 mL of MRS broth for *L. jensenii* and *L. crispatus* or TS broth for *S. epidermidis* and cultured at 37°C for 18 h in a sealed 15 mL conical tube (BD). After 18 h, the number of viable bacteria present was quantified by plating of serial 10-fold dilutions onto MRS or TS agar plates (BD) or by the liquid-based quantification assay. For the liquid-based quantification assay, MRS or TS broth (90 uL) was added to all wells of a 96 well culture plate (BD) and 10 uL of the bacterial stock was added to the first column. Serial 10-fold dilutions into the remaining 11 columns were performed subsequently. The plates were incubated for 24 h then visually inspected using an Axiovert 25 microscope (Zeiss, Thronwood, NY) at 20× magnification to identify the last well that contained bacteria. Based on these serial dilutions, the approximate cfu/mL of each bacteria present in the tubes after 18 h of growth was calculated. This system also was used for quantifying bacterial loads in the VEC multilayer cultures through triplicate platings. Standard agar plating was directly compared to the liquid-based quantification to confirm the validity of this higher-throughput approach (Figure 3A).

To prepare *L. iners*, a frozen stock was thawed and then cultured for 48 h on chocolate agar plates anaerobically. After 48 h, the bacteria were scraped from the plate into sterile PBS, and the amount of viable bacteria present was quantified using serial dilutions on chocolate agar plates. All bacteria were pelleted and washed extensively before final suspension into KSFM prior to VEC inoculation.

To quantify long-term bacterial viability in the multilayer cultures and the impact of bacteria on VEC health, 1000 cfu of selected bacteria (10 uL) or KSFM (10 uL) was added to the apical air-interface. The cultures were incubated for up to 5 d and at selected times 50 uL of KSFM was added to the top chamber followed by mechanical disruption of the multilayer. After mixing to create a suspension of material, two 10 uL aliquots of the sample were subjected to bacterial titration using the broth-based viability analysis. The remaining 30 uL of sample was analyzed via MTT assay using 1 mg/mL Thiazolyl Blue Tetrazolium Bromide to assess cell health (Sigma, St. Louis, MO). MTT values were collected on a Versamax microplate reader (560 nm; Molecular Devices; Sunnyvale, CA). Changes in apical cell surface pH were measured in triplicate wells using pH 4.0–8.0 indicator strip material (EMD, Gibbstown, NJ) at 6, 18 and 24 h post addition of the bacteria.

### Histological Staining and Immunofluorescent Labeling

VEC multilayer cultures (d 9) were fixed with Z-fix (Anatech, Battle Creek, MI) for 24 h at 4°C. The filters were excised, paraffin embedded and 5 um sections were cut using a Microm HM 310 microtome (Fisher, Pittsburgh, PA) prior to mounting on slides. The sections were hematoxylin and eosin (H&E) or periodic acid Schiff (PAS) stained to visualize the cell layers or glycogen deposits, respectively. For confocal observation of multilayer formation, the cultures were Z-fixed and washed 3 times with tris-buffered saline (Fisher) then the filters were excised and mounted on slides. Cells were labeled using Vectashield with DAPI (Vector Laboratories, Burlingame, CA) prior to visualization on a LSM 510 UV Meta Laser Scanning Confocal Microscope (Zeiss).

To observe bacterial colonization of the apical cell surface, 1000 cfu of selected bacteria were added to d9 GFP-expressing V19I cultures. After 24 or 72 h the fixed culture inserts were labeled with a monoclonal antibody recognizing the lipoteichoic acid of Gram+ bacteria (clone 3801; Santa Cruz Biotechnology, Santa Cruz, CA) and a goat anti-mouse secondary conjugated to AF555 (Invitrogen). Filters were removed, mounted using Vectashield with DAPI and visualized by confocal microscopy.

### Transmission, Immunogold Occludin-Labeling and Scanning Electron Microscopy

VEC multilayer cultures (d 9) 24 h post addition of medium controls or 1000 cfu of selected bacteria were Z-fixed and used for electron microscopy (EM) experiments. Cultures were rinsed with cacodylate buffer, postfixed in 1% OsO4 in 0.1 M cacodylate buffer for non immunogold EM samples, rinsed with water, stained en bloc with 2% aqueous uranyl acetate and dehydrated in ethanol. Filters were excised and embedded in Poly-Bed 812 resin (Polysciences Inc., Warrington, PA) or LR White (Electron Microscopy Sciences, Hatfield, PA) for immunogold EM samples. Ultrathin sections were cut on a Leica EM UC7 ultramicrotome (Leica Microsystems, Bannockburn, IL) then labeled with rabbit anti-human occludin (Santa Cruz Biotechnology, Santa Cruz, CA) and a 15 nm gold-labeled goat anti-rabbit IgG (Aurion, Electron Microscopy Services). Sections were stained with lead citrate then examined on a Philips CM100 TEM at 60 kV.

For scanning EM, the cultures were fixed as above then dehydrated in ethanol and processed through hexamethyldisalazane followed by air-drying. The filters were excised, mounted onto metal stubs and sputter-coated with iridium in an Emitech K575× Sputter Coater (Emitech, Houston, TX) at 20 mA for 20 sec. The filters were examined in a Hitachi S4700 SEM (Hitachi High Technologies, Schaumburg, IL) at 2 kV.

### TLR Agonists and Cytokine Quantification

Synthetic TLR agonists (Invivogen; San Diego, CA) tested were peptidoglycan (PGN, TLR2), fibroblast stimulating ligand-1 (FSL-1, TLR2/6) and polyinosinic∶polycytidylic acid (PIC, TLR3). Recombinant *Salmonella dublin* flagellin (FLAG, TLR5) was produced and purified as previously described [57]. For TLR agonist treatments, VEC multilayer cultures were incubated for indicated periods of time with 1000 cfu of selected bacteria or KSFM. After 24 h, PIC (0.1 mg/mL), FSL-1 (0.1 ug/mL), FLAG (5 ug/mL), PGN (0.1 mg/mL) or KSFM (10 uL) was added to the apical surface. After 6 or 24 h, 100 uL of medium was added to the apical surface and gently moved over the surface by pipeting simulating a vaginal lavage before being collected and stored at −80°C. Culture lavages (50 uL) were evaluated using the BioPlex Human Group I cytokine kit (Bio-Rad, Hercules, CA) following the recommendations of the manufacturer. Absolute cytokine quantities (pg/mL) were extrapolated from a standard curve run in parallel.

### Statistical Analyses

Data were analyzed for significance with the Student's t-test using the Prism software package v4.0 (Graph Pad, San Diego, CA). Each study was repeated at least once with triplicate samples to confirm results.
